# Normative evolution and institutional challenges of reproductive health of trans men and transmasculine people in Brazil: a scoping review

**DOI:** 10.1590/0102-311XEN200525

**Published:** 2026-07-27

**Authors:** Nicole Delgado Austregésilo, Danielle Boachá Sampaio, Marina Sales Afonso de Melo, Vitória Camila Lima Mello de Moraes Marques, Eduarda Correia Moretti, Caroline Wanderley Souto Ferreira, Leila Maria Alvares Barbosa

**Affiliations:** 1 Universidade Federal de Pernambuco, Recife, Brasil; 2 Universidade Federal de Alagoas, Maceió, Brasil.

**Keywords:** Reproductive Health, Transgender Persons, Gender Identity, Reproductive Rights, Saúde Reprodutiva, Pessoas Transgênero, Identidade de Gênero, Direitos Sexuais e Reprodutivos, Salud Reproductiva, Personas Transgénero, Identidad de Género, Derechos Sexuales y Reproductivos

## Abstract

This study aimed to map and analyze the normative evolution and institutional challenges related to the reproductive health of trans men and transmasculine people in Brazil. A scoping review was carried out according to the methodological guidelines of the JBI, registered on the Open Science Framework. The retrieved scientific studies included articles, theses, dissertations, academic papers, and various official regulations. The findings show that, despite important legal frameworks, these regulations emerged in historical contexts in which the identity categories and specific demands of trans men and transmasculine people were not yet consolidated in the public and legal debate. Notably, by remaining without substantive updates, these provisions produce effects of exclusion and invisibility, showing the persistence of a cisheteronormative logic in the structuring of access to reproductive health. Recent advances include the recognition of trans parenting in official documents and the creation of specific materials, such as the *Transgesta* prenatal booklet. However, critical gaps persist in access to fertilization, gamete preservation, and family planning, as do symbolic and institutional barriers to health care. This study shows that the fulfilment of sexual and reproductive rights of this group depends on more inclusive normative frameworks and on the transformation of institutional practices and the training of healthcare providers. The historical and critical analysis of policies in this study evince advances, setbacks, and omissions, contributing to expand knowledge about the reproductive health of trans men and transmasculine people in Brazil.

## Abstract (pt)

Este estudo teve como objetivo mapear e analisar a evolução normativa e os desafios institucionais relacionados à saúde reprodutiva de homens trans e pessoas transmasculinas no Brasil. Uma revisão de escopo foi realizada de acordo com as diretrizes metodológicas do JBI, registrado no Open Science Framework. Foram localizados estudos científicos, incluindo artigos, teses, dissertações, artigos acadêmicos e diversas regulamentações oficiais. Os achados revelam que, embora existam marcos legais importantes, essas regulamentações foram formuladas em contextos históricos onde as categorias de identidade e as demandas específicas de homens trans e pessoas transmasculinas ainda não estavam consolidadas no debate público e jurídico. No entanto, ao permanecerem sem atualizações substanciais, essas disposições produzem efeitos de exclusão e invisibilidade, revelando a persistência de uma lógica cisheteronormativa na estruturação do acesso à saúde reprodutiva. Avanços recentes incluem o reconhecimento da parentalidade trans em documentos oficiais e a criação de materiais específicos, como o livreto pré-natal *Transgesta*. No entanto, persistem lacunas críticas no acesso à fertilização, preservação de gametas e planejamento familiar, além de barreiras simbólicas e institucionais ao cuidado da saúde. O estudo mostra que a realização dos direitos sexuais e reprodutivos desse grupo depende não apenas de estruturas normativas mais inclusivas, mas também da transformação das práticas institucionais e da formação de profissionais de saúde. Concluiu-se que a análise histórica e crítica das políticas nos permite compreender avanços, retrocessos e omissões, contribuindo para ampliar o conhecimento sobre a saúde reprodutiva de homens trans e pessoas transmasculinas no Brasil.

## Abstract (es)

Este estudio tuvo como objetivo mapear y analizar la evolución normativa y los desafíos institucionales relacionados con la salud reproductiva de hombres transgénero y de personas transmasculinas en Brasil. Se realizó una revisión de alcance de acuerdo con las directrices metodológicas del JBI, registradas en el Open Science Framework. Se localizaron estudios científicos, incluidos artículos, tesis, disertaciones, trabajos académicos y diversas reglamentaciones oficiales. Los hallazgos revelan que, si bien existen importantes hitos legales, estas reglamentaciones se formularon en contextos históricos en los que las categorías de identidad y las demandas específicas de los hombres trans y de las personas transmasculinas aún no estaban consolidadas en el debate público y jurídico. Sin embargo, al no haber sido actualizadas sustancialmente, estas disposiciones producen efectos de exclusión e invisibilidad, lo que revela la persistencia de una lógica cisheteronormativa en la estructuración del acceso a la salud reproductiva. Entre los avances recientes se incluyen el reconocimiento de la paternidad transgénero en documentos oficiales y la creación de materiales específicos, como el folleto prenatal *Transgesta*. Sin embargo, persisten importantes deficiencias en el acceso a la fertilización, a la preservación de gametos y a la planificación familiar, además de barreras simbólicas e institucionales al cuidado de salud. El estudio demuestra que el ejercicio de los derechos sexuales y reproductivos de este grupo depende no solo de estructuras normativas más inclusivas, sino también de la transformación de las prácticas institucionales y de la formación de profesionales de la salud. Se concluyó que el análisis histórico y crítico de las políticas permite comprender los avances, los retrocesos y las omisiones, lo que contribuye a una comprensión más amplia de la salud reproductiva de los hombres transgénero y de las personas transmasculinas en Brasil.

## Introduction

Reproductive health is an essential human right that is directly associated with dignity, autonomy and citizenship. According to the World Health Organization (WHO), this complete state of physical, mental, and social well-being includes all aspects related to the reproductive system and its functions beyond the absence of diseases ^1^.

The 1948 *Universal Declaration of Human Rights* introduced the principles of dignity and equality for all people ^2^. Then, the Cairo International Conference on Population and Development (held in Egypt) marked a turning point by explicitly linking health, sexuality, and reproductive rights, incorporating debates on gender inequalities ^1^. The Fourth World Conference on Women, held in Beijing (China) in 1995, consolidated the vision of sexual and reproductive rights as part of universal human rights ^3^. In 2007, the *Yogyakarta Principles* went even further by establishing specific guidelines for the application of international human rights law regarding sexual orientation and gender identity ^4^.

The 1988 *Brazilian Federal Constitution* guarantees the right to family planning, giving the State the responsibility to guarantee access to information and contraceptive methods ^5^. *Law n. 9,263/1996* ^6^ regulated this right, attributing to the Brazilian Unified National Health System (SUS, acronym in Portuguese) the provision of assistance for conception and contraception. However, its effectiveness remains uneven, especially among marginalized populations, such as trans men and transmasculine people (encompassing trans men on the binary spectrum and people assigned female at birth who identify on the nonbinary spectrum of transmasculinity), who remain on the margins of public policies.

These people often face institutional and social barriers rooted in a cisheteronormative logic that disregards their needs, such as the guarantee of the right to family planning and pregnancy ^7^. Understanding the current difficulties in access to reproductive health services by trans men and transmasculine people requires an analysis of the historical and legal path that has structured the sexual and reproductive rights of this portion of the population of lesbian, gay, bisexual, transsexual, and other (LGBT+) people.

In view of this scenario, this study aims to map and analyze the normative evolution and institutional challenges related to the reproductive health of trans men and transmasculine people in Brazil. For this, a scoping review was carried out with a focus on legal, regulatory, and political frameworks that partially or fully address the reproductive rights of this population. It aims to understand the historical path of legal guarantees and public policies in this field, highlighting the advances, omissions, and contradictions in the formulation and implementation of these norms to contribute to critical knowledge that subsidizes more equitable and inclusive practices and policies within SUS.

## Methods

The scoping review was conducted from February to September 2025, following the JBI methodology and the standards of the *Preferred Reporting Items for Systematic reviews and Meta-Analyses extension for Scoping Reviews* (PRISMA-ScR) checklist ^8^ (Supplementary Material; https://cadernos.ensp.fiocruz.br/static//arquivo/suppl-e00200525_2251.pdf). The review protocol has been registered on the Open Science Framework (DOI: https://doi.org/10.17605/OSF.IO/NGYVM).

The objective of the research was structured based on the PCC strategy: Population (transgender men and transmasculine people), Concept (reproductive health), and Context (Brazilian law), as proposed by Peters et al. ^9^. The following guiding questions were formulated based on this framework: which Brazilian legislative frameworks address the reproductive health of transgender men and transmasculine people? What are the legal implications for this group’s access to reproductive health, especially regarding fertilization, gamete preservation, and family planning? How do SUS regulations address the specificities of reproductive care for transgender men and transmasculine people? Are there specific public policies to ensure access to reproductive health services without discrimination or violation of the rights of this group?

In this study, normative evolution refers to the changes over time in the legal and political recognition of the reproductive rights of trans men and transmasculine people, considering the existence or absence of legal, regulatory, and programmatic instruments and the transformations in the language and categories mobilized by these norms. Institutional challenges concern the barriers faced by these individuals in exercising their reproductive rights due to normative, organizational, and implementation limitations within State institutions, especially the SUS.

### Eligibility criteria

Official documents (primary sources - laws, decrees, administrative ordinances, resolutions, technical notes, national plans, and public policies) and scientific studies (secondary sources) that addressed in whole or in part the reproductive health of trans men and transmasculine people in Brazil were included. Studies that did not analyze the three aspects of the PCC strategy were excluded.

The searches were conducted on April 24, 2025. The legal and governmental databases (Planalto portal ENT#091;https://www.gov.br/planalto/pt-brENT#093;, *Official Gazette of the Union* ENT#091;DOU - Diário Oficial da UniãoENT#093;, and Brazilian Ministry of Health) were selected based on three criteria: (i) the sphere of normative production (especially the federal level), responsible for formulating the main guidelines and policies of the SUS; (ii) the type of document - the Planalto portal was used for laws and decrees, the DOU, for normative and administrative acts (such as ordinances, resolutions, and notices), and the Brazilian Ministry of Health, for public policies, guidelines, and technical materials; and (iii) the feasibility of access, considering the lack of a single, systematized database for retrieving regulations in Brazil and the reliability, public access, and continuous updating of these sources. The following scientific databases were selected: Scopus, MEDLINE/PubMed, Google Scholar, Embase, and LILACS. The grey literature and reference lists of the included studies were also examined for additional relevant materials.

### Search strategy

Due to the lack of standardized indexing, searches in legal and institutional databases were conducted using keywords that were individually applied to each search: “trans man”, “transmasculine person”, “transgender”, “transsexuals”, “person who gestates”, “pregnant person”, and “person with a uterus” in Portuguese. The found documents were analyzed for the following themes: “reproductive rights” and “reproductive health”. Additionally, normative documents focusing on reproductive health were examined to verify whether they explicitly or indirectly addressed the target population.

The search strategies (Supplementary Material; https://cadernos.ensp.fiocruz.br/static//arquivo/suppl-e00200525_2251.pdf) used in the academic databases were developed from MeSH (*Medical Subject Headings*), DeCS (*Descritores em Ciências da Saúde*), and Emtree descriptors, including equivalents in English and Portuguese to terms such as “trans men”, “transmasculine people”, “reproductive health”, and “reproductive rights”; used in tandem with the Boolean operators “AND” and “OR”. The research was conducted using the “all fields” search field, enabling the retrieval of these terms in the title, abstract, and other fields of the bibliographic record.

### Documents selection

The normative documents were chosen based on an analysis of their full content since these materials generally have no structured abstracts. Scientific studies were selected using the Rayyan (https://www.rayyan.ai/). Then, the titles, abstracts (when available), and full content of the retrieved articles were sequentially read in full. No hierarchy between primary and secondary sources was used; both were selected concurrently according to previously defined eligibility criteria. This step was performed independently by two reviewers (N.D.A. and D.B.S.), and disagreements were resolved by consensus.

Considering the nature of the guiding questions in this research (which involve the identification of normative frameworks and the analysis of their legal, institutional, and operational implications), primary and secondary sources were complementarily integrated. Primary sources constituted the central axis of the analysis, being used to map, describe, and systematize the current normative framework. Academic literature, in turn, was mobilized as analytical support, enabling the critical interpretation of these documents, including aspects related to their implementation, regulatory gaps, institutional contradictions, and users’ and healthcare providers’ experiences. This strategy is consistent with the exploratory nature of scoping reviews, which can articulate several types of evidence in complex analytical contexts.

### Data extraction and analysis

Data were independently extracted by two reviewers (N.D.A. and D.B.S.) using a standardized form developed for this review. The normative documents were listed and organized by institutional repository and chronological order, including information on document type, publication/update date, responsible body, main content, and specificities. The following variables were extracted from the scientific studies: authors, year, title, document category, study design, objective, and main results.

The extracted data were organized into a structured spreadsheet and subjected to descriptive synthesis and thematic analysis. The identified normative frameworks were first systematized in chronological order to map their evolution throughout the analyzed period. Next, the content of the scientific studies was thematically coded, highlighting “advances” and “challenges”.

The findings in the analytical process showed linear organization, traversing normative, institutional, and operational dimensions in an interdependent manner. Given this complexity, the data were reorganized into analytical thematic axes that were inductively constructed based on the critical analyses presented by the authors and the reports of users and healthcare providers in the qualitative studies. This step enabled the articulated integration of aspects related to normative advances, regulatory gaps, institutional contradictions, the use of inclusive language, and the implementation of policies within SUS.

## Results

The legal and governmental databases included documents such as laws, decrees, resolutions, administrative ordinances, public notices, technical notebooks, booklets and other informative materials. These documents formed the basis for the identification and systematization of current regulatory frameworks, being analyzed in conjunction with findings from the academic literature, which furthered the understanding of their formulation, implementation, and limitations.

**Figure 1:**
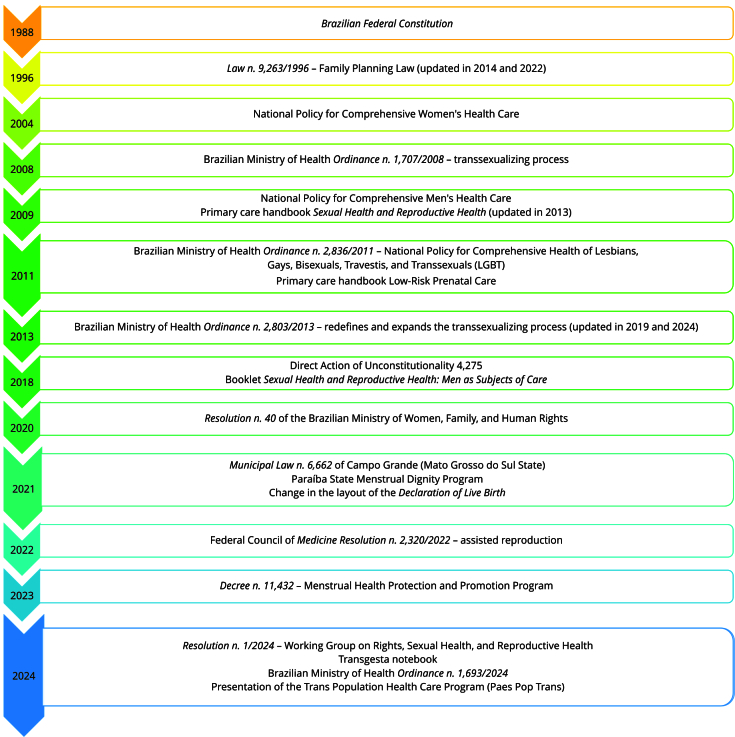
Timeline of the main Brazilian regulatory frameworks related to reproductive health and the health of the LGBT+ population, 1988-2024.

Source: prepared by the authors.

Of the 21 included normative documents, this research retrieved some of them by searches in legal and governmental databases, finding others from the analyzed academic studies (i.e., not by a direct search). This complementarity highlights the operational limitations of search strategies in institutional repositories and reinforces the importance of integration between sources. Figure 1 describes the chronological organization of these milestones, illustrating their evolution over time.

The limitations of search systems in legal and governmental databases prohibited the determination of the total number of retrieved records in the initial stage since these platforms neither provide the complete quantity of results nor enable the export or traceability of the performed strategies. Thus, the PRISMA-ScR flowchart in this study ignored normative documents, only representing the eligible scientific studies.

**Figure 2:**
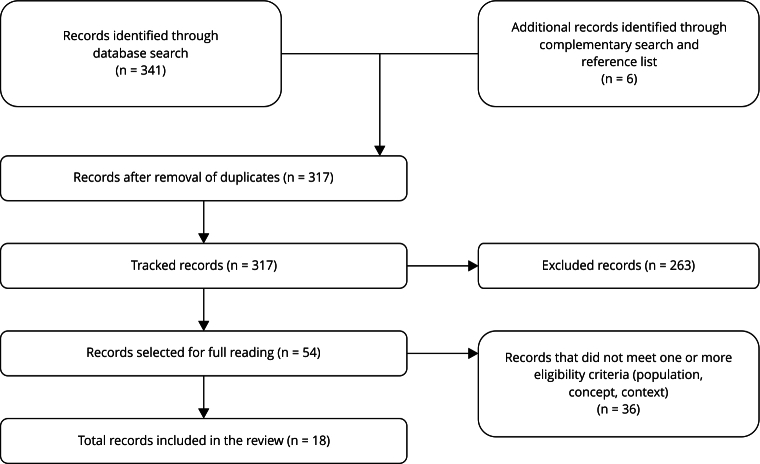
Flowchart of search and selection of scientific studies addressing the normative evolution and institutional challenges related to the reproductive health of trans men and transmaculine people in Brazil, 2019-2025.

Source: prepared by the authors.

The search and selection of scientific studies followed the strategy of the PRISMA-ScR flow diagram (Figure 2). This study retrieved 341 documents from MEDLINE/PubMed (n = 6), Embase (n = 21), Scopus (n = 10), Web of Science (n = 5), LILACS (n = 9), and Google Scholar (n = 290). In total, 12 met the set eligibility criteria. The complementary search in reference lists and institutional repositories found and included another six documents, totaling 18 analyzed documents.

Of the included studies, 10 were published in scientific journals, three were final papers, two were master’s dissertations, two were PhD theses, and one was a book chapter. Box 1 shows the main information from these studies, including author, year of publication, title, category of the document/study design, and objective. The included studies were mostly published from 2015 to 2025, emphasizing themes related to access to reproductive health, service organization, and the sexual and reproductive rights of trans men and transmasculine people. Qualitative, documentary, and exploratory approaches in several fields of knowledge (such as public health, law, nursing, and social sciences) predominate, highlighting the interdisciplinary nature of the topic and the complexity of its dimensions.

**Box 1:** Characteristics of included studies addressing normative evolution and institutional challenges related to the reproductive health of trans men and transmasculine people in Brazil, 2019-2025.

STUDY (YEAR)	TITLE *	DOCUMENT CATEGORY STUDY DESIGN	OBJECTIVE
Holanda ^28^ (2019)	*For the Affirmation of the Right to Homosexual and Transsexual Filiation Resulting from Assisted Human Reproduction Techniques in Brazil: From Favorable Judicial Decisions Towards the Necessary Legislation*	Dissertation (Law) Legislative analysis (legal, sociological, anthropological and bibliographical assessment) zetetic approach	Construct legal arguments based on doctrine, jurisprudence and fundamental rights to defend the legal regulation of the right to filiation through assisted human reproduction techniques (ART) in same-sex and transsexual families in Brazil
Vicente ^22^ (2020)	*Sexual and Reproductive Rights of Trans Men,* boycetas *and Nonbinary People: A Struggle for Recognition and Redistribution of Public Health in Brazil*	Monograph (Public Administration) Autoethnographic research, interviews with professionals, researchers and activists in the field, trans and nonbinary men who are pregnant or have pregnant	Understand whether the SUS seeks to meet the sexual and reproductive rights of trans men, *boycetas* and nonbinary people with vaginas
Natividade ^40^ (2021)	*Reconstructing Centers and Margins: Research Notes for Assessing Sexual Politics in Contemporary Brazil*	Article Document analysis	To assess recent setbacks in Brazilian sexual politics in the face of the rise of conservative and fundamentalist agendas, especially in the post-impeachment context of Dilma Rousseff and during the Jair Bolsonaro administration
Rotondano et al. ^25^ (2021)	*Changing the Civil Registry of Transgender People Without the Need for Sex Reassignment Surgery in Brazil*	Article Bibliographic, documentary, comparative, historical and qualitative research, using inductive and dialectical methods	To evaluate the possible advances of Direct Action of Unconstitutionality 4,275/DF in the scenario of subordination of transgender people in Brazil
Okano ^19^ (2022)	*Comprehensive Care for the Trans Population: What is the Role of Primary Health Care (PHC)?*	Article Epidemiological bulletin	Informative/Educational guide to guide health professionals in caring for trans people in PHC
Okano et al. ^39^ (2022)	*Reproductive Care for Transgender People: From Pregnancy Planning to the Postpartum Period - A Narrative Review*	Article Narrative review	Evaluate pregnancy planning and contraception; the possibility of using assisted reproduction techniques according to the recommendations of the Federal Council of Medicine in Brazil; and pregnancy, prenatal care and postpartum care in the transgender population
Pereira ^15^ (2022)	*Social Representations of Pregnancy among Trans Men*	Dissertation (Nursing) Qualitative, descriptive and exploratory study, anchored by the Theory of Social Representations	To understand the meanings and experiences of pregnancy among trans men
Xavier ^30^ (2022)	*From Poverty to Menstrual Dignity: An Analysis of Legislation and Public Policies in Brazil*	Monograph (Law) Historical analysis, conceptualization, international scenario and analysis of Brazilian legislation	Understand menstrual poverty, its concept and impacts
Martimiano ^12^ (2023)	*Transmasculinities and Pregnancy: An Analysis of Public Health Policies*	Monograph (Psychology) Documentary analysis and exploratory phase/fieldwork	Understand how pregnancy and gestation of transmasculine people appear in three previously mentioned and presented SUS health policies, considered constitutive of the reproductive health of the LGBTQIA+ population
Nascimento ^36^ (2023)	*Reproductive Rights and Access to Assisted Reproduction for Transmasculine People and Trans Men in Brazil - Public Policies, Medical Perspectives and Trans Experiences*	Thesis (Public Health) Exploratory descriptive research with a qualitative approach, carried out through the triangulation of data sources	To analyze reproductive rights and access to assisted reproduction for transmasculine people in Brazil, encompassing public policies, medical perspectives, and those of transmasculine people, including their needs and expectations regarding biological parenthood
Santos ^29^ (2023)	*Dissident Parenting: Caregiving by Trans Men in Brazil and Portugal*	Thesis (PhD in Feminist Studies) Qualitative, comparative study	To investigate the parenting experiences of trans men in Brazil and Portugal, in conjunction with the frameworks of intimate, sexual, and reproductive citizenship. The research seeks to understand the legal and social support mechanisms and public policies involved in recognizing these parenthoods and guaranteeing their rights
Yoshioka & Oliveira ^10^ (2023)	*Obstetric Violence and the Vulnerability of Trans Men Regarding their Personality Rights in Brazilian Healthcare Services*	Article Theoretical, documentary research and hypothetical-deductive method	To assess the impacts of transphobia on obstetric violence suffered by transgender men in Brazilian health services in the 21st century
Freitas et al. ^41^ (2024)	*Social Movements and the Genesis of Proposals for the Health of the LGBT Population in Bahia, Brazil (1979-2014): Initial Disputes and Possible Alternatives*	Article Study partner history	To describe the genesis of health proposals aimed at the LGBT population in the state of Bahia between 1979 and 2014, based on the sociological analysis of public policies according to Patrice Pinell’s approach
Lemos ^14^ (2024)	*Public Policies on Pregnancy, Practices and Discursive Experiences of Transmasculine Pregnancy*	Book chapter	Describe health policies and their absence in relation to the reproductive rights of transmasculine people who do, or do not, undergo transsexualizing and externalizing processes during pregnancy, childbirth and breastfeeding
Mascarenhas et al. ^35^ (2024)	*Trans Man and Paternal Pregnancy: Experiences During the Pregnancy-Puerperal Period*	Article Qualitative, narrative and reflective case study of everyday professional experiences	To analyze the experiences of a trans man during the pregnancy-puerperal period and the perspective of obstetric nurses in training, based on the dynamics and organization of obstetric health care in a hospital environment
Prado ^32^ (2024)	*Public Policies on Menstrual Health in Brazil: Perspectives through the Lens of Social Movements Related to Menstruation*	Article Narrative review and documentary analysis	Conduct a survey of public policies for women’s health, bills on menstrual dignity and the work of social movements related to menstruation in Brazil
Silva et al. ^24^ (2024)	*Transgender Men and Pregnancy: An Integrative Literature Review*	Article Integrative review	To understand the current evidence on pregnancy in transgender men in the context of sexual and reproductive health care, focusing on the organization of health services and the experiences of these individuals during the pregnancy-puerperal cycle
Avelino et al. ^16^ (2025)	*Transmasculinities, Pregnancy and Support in the Healthcare System*	Article Qualitative study with an ethnographic or participatory approach, with elements of action research	To reflect on the implications of transmasculine pregnancy for health policies and services, based on intervention research in a SUS outpatient clinic in Northeast Brazil

PHC: primary health care; SUS: Brazilian Unified National Health System.

* Free translation from the original Portuguese titles.

In general, such production shows a field still under consolidation, in which advances in the normative recognition of rights coexist with regulatory gaps and operationalization weaknesses. Challenges related to the implementation of policies within health services stand out in this context, including institutional barriers, inadequacies in normative language, and limitations in access to reproductive care.

Box 2 summarizes the main findings of this review, organizing themes/rights, their corresponding normative instruments, and the found advances and limitations. This summary stems from an integrated analysis of the included normative documents and scientific studies, articulating legal frameworks with their practical and institutional implications.

**Box 2:** Summary of themes related to the reproductive health of trans men and transmasculine people in Brazil, indicating the corresponding regulatory frameworks, advances, and limitations in the integrated analysis of regulatory documents and scientific studies, 2019-2025.

RECOGNIZED THEME/LAW	RELATED DOCUMENTS	ADVANCES	LIMITATIONS
Family planning	*Brazilian Federal Constitution* (1988), *Law n. 9,263/1996*	Access by SUS	Cisheteronormative language
Reduction of morbidity and mortality feminine	National Policy for Comprehensive Women’s Health Care, Alyne Network	Safe abortion and combating domestic and sexual violence	Cisheteronormative language
Co-responsibility of men	National Policy for Comprehensive Men’s Health Care	-	Unpreparedness of health services and institutional violence
Transsexualizing process	*Ordinance n. 1,707/2008* and *Ordinance n. 2,803/2013* (Brazilian Ministry of Health)	Increased comprehensiveness of care	Does not mention reproductive health
Basic attention notebooks​	*Sexual and Reproductive Health* and *Low-Risk Prenatal Care*	Recognizes the vulnerability of trans people	Cisheteronormative language
Health of the LGBT population	National Policy for Comprehensive Health of Lesbians, Gays, Bisexuals, Travestis, and Transsexuals	Recognizes the demands of this population	Does not mention reproductive rights of trans men
Use of social name	Direct Action of Unconstitutionality 4,275	Right to change name and gender in civil registry	Disrespect and use of deadname
Menstruation	*Resolution n. 40/2020* (Brazilian Ministry of Women, Family, and Human Rights), *Municipal Law n. 6,662/2021* (Campo Grande, Mato Grosso do Sul State), *Decree n. 11,432/2023*	Menstrual Health Protection and Promotion Program, includes “people who menstruate”	Outdated other documents and barriers to access
Assisted reproduction	*Resolution n. 2,320/2022* (Federal Council of Medicine)	Provided an opportunity for other settings with family members	Backtracking on removing “transgender people” from the text
Transparency	*Declaration of Live Birth*	Recognition of pregnancies of trans men	Discrimination and inappropriate behavior of healthcare providers
Materials informative	Booklets *Sexual Health and Reproductive Health: Men as Subjects of Care* and *Health of Trans Men and Transmasculine People*	Prepared by the transmasculine population itself	It underwent changes by the current government, characterizing a setback
Transsexuality	International Classification of Diseases (ICD) - 11th edition	Removal from the list of mental illnesses	-

SUS: Brazilian Unified National Health System.

Source: prepared by the authors.

The findings show that normative, institutional, and operational aspects are interconnected, creating a field marked by tensions between the formal recognition of rights the and limitations in their implementation. This complexity guides the analysis in the following section.

## Discussion

An integrated analysis of normative documents and scientific studies shows the complex and interdependent evolution of reproductive health for transgender men and transmasculine individuals in Brazil, encompassing normative, institutional, and operational dimensions. This context shows progress in the recognition of rights but also regulatory gaps and persistent challenges in their implementation. Therefore, this study discusses this theme from a critical chronological perspective, seeking to highlight how these processes were constituted and how tensions arose over time.

### Generic policies and initial legal recognition

By adopting the dignity of the human person as one of its fundamental principles, the 1988 *Brazilian Federal Constitution* recognizes that personality rights are innate and inherent to human beings. Thus, they require recognition even if not provided for by law ^5^. This perspective recognizes sexual and reproductive rights (even without their express provisions in the *Federal Constitution*) as personality rights based on the dignity of the human person and the right to free family planning ^10^.

*Law n. 9,263/1996*, the Family Planning Law, constitutes the main Brazilian norm to regulate reproductive rights. It establishes principles and guarantees that ensure all people the right to exercise their sexuality, decide on the number of children and the interval between pregnancies, and guarantees access to contraceptive methods and voluntary sterilization at SUS. However, the law has significant gaps regarding the reproductive rights of trans men and transmasculine people in its original wording and in its 2014 and 2022 updates ^6^ ^,^ ^10^. Its language is predominantly heteronormative and centered on the figure of the “couple” (generally interpreted as the union between a cisgender man and a woman), having no mention of trans people or explicit recognition of dissident family arrangements.

In 2004, the Brazilian Ministry of Health created the National Policy for Comprehensive Attention to Women’s Health (PNAISM, acronym in Portuguese) ^11^. Promoting women’s autonomy and aiming to reduce female morbidity and mortality, it outlined health actions focused on family planning, obstetrics, safe abortion, and the fight against domestic and sexual violence ^12^. The policy document (included in the Federal Government’s Multiannual Plan for 2024 to 2027) and “Alyne Network” (formerly “Stork Network”) consider that the bodies that can become pregnant are exclusively cis and straight women, showing negligence and advances in policies that include trans men and transmasculine people ^13^ ^,^ ^14^ ^,^ ^15^.

Similar to the PNAISM, the 2009 National Policy for Comprehensive Men’s Health Care (PNAISH, acronym in Portuguese) also received criticism. First, its document bringing men closer to co-responsibility in reproductive planning creates a distance from the reality of trans men’s pregnancies ^12^. The policy ignores these experiences, rendering them invisible and perpetuating the unpreparedness of health services and institutional violence ^16^. At the same time, its objectives include “*promoting comprehensive men’s health care in travesti and transsexual populations*” denoting a conception of gender based on biological determinism ^15^.

The Brazilian Ministry of Health *Ordinance n. 1,707/2008* ^17^ set a major milestone for the health of the trans population by instituting the transexualizing process at SUS, which the Brazilian Ministry of Health *Ordinance n. 2,803/2013* ^18^ redefined and expanded, increasing the comprehensiveness of care from primary care to tertiary services responsible for hormonal and surgical interventions. Also, two updates occurred in 2019 and 2024, including new surgical procedures. However, there remains no mention of the preservation of users’ fertility ^10^ ^,^ ^19^. Lemos ^14^ states that the perception of cisgender people about transgender people seeking the transexualizing process is of “wanting to be a cis person”, denying them their reproductive rights. Other authors point to the poor conduct of healthcare providers in failing to guide them regarding the impairment of their reproductive capacity when performing certain procedures and in discussing the possibilities of fertility preservation methods, generating what is known as “symbolic castration” ^10^ ^,^ ^14^ ^,^ ^15^.

The primary care notebooks the Brazilian Ministry of Health prepared include the *Sexual Health and Reproductive Health* booklets (published in 2009 and updated in 2013) ^20^ and the *Low-risk Prenatal Care* booklet (published in 2012) ^21^. In the first, the government clearly recognizes the vulnerability and violation of human rights faced by trans people, which directly affects their conditions to freely exercise their reproductive practices if they wish ^15^. The Brazilian Ministry of Health also admits that its measures mostly target cis women. However, despite the detailed chapter on the LGBT+ population (which states that prejudice and discrimination configure factors that make people vulnerable, which requires considering them as determinant and conditioning factors of the health of this population), the Brazilian Ministry of Health fails to mention of trans men ^22^. Meanwhile, the second notebook includes a totally cisheteronormative language.

The National Comprehensive Health Policy for Lesbians, Gays, Bisexuals, Travestis, and Transsexuals, instituted by the Brazilian Ministry of Health *Ordinance n. 2,836/2011* ^23^, has established itself as a turning point for public health policies in Brazil, recognizing the demands of this population. Although it seeks to ensure respect for free sexual orientation and gender identity and to recognize all configurations of family constitution, it has no specific mention of the reproductive rights of trans men ^10^ ^,^ ^24^. Thus, implementing the policy guidelines, reducing the faced challenges, ensuring access, and encompassing the reality of transmasculine people require updates and reformulations ^15^ ^,^ ^16^ ^,^ ^22^.

### Legal advances in identity recognition

The recognition of a person’s identity (especially by the name with they identify) is a fundamental condition for the full exercise of individuals’ rights. The lack of such recognition imposes symbolic and institutional barriers from their first contact with public services, making dignified and equal access to health unfeasible. In the case of trans men and transmasculine people, the social name is no longer just an administrative matter, representing the minimum respect necessary for access to any right ^25^.

The Brazilian Ministry of Health regulated the use of the social name in users’ documents in 2009. Over the years, several regulations in various spheres (such as tests, competitions, and public offices) have incorporated it ^25^. However, only in 2014, when the importance of drawing attention to violent crimes against the LGBT population was established, that parameters were established to include the following items in police reports: “sexual orientation”, “gender identity”, and “social name” ^26^. Moreover, the Brazilian Federal Supreme Court (STF, acronym in Portuguese) only decided that homophobia and transphobia are crimes (equating them to racism) in 2019. They based their decision on the Racism Law (*Law n. 7.716/1989*). Later, the Brazilian National Congress created a specific law on the subject ^27^.

The 2018 Direct Action of Unconstitutionality 4,275 was an important legal milestone for transgender people since it recognized their right to change their name and gender in their civil registry regardless of sex reassignment surgery. This decision was based on their difficulties in accessing surgical procedures at SUS and especially on the autonomy of trans people to determine their gender, even if they neither want nor need surgery to have it validated ^25^. In this context, self-determination, as a constitutional basis for the exercise of parenthood or “*genitorialidade*”, proposed by Holanda ^28^, is substantial to the reproductive freedom and family planning of transmasculine people, which requires institutional reforms that respect bodily integrity and reproductive rights ^16^ ^,^ ^25^ ^,^ ^28^ ^,^ ^29^.

### Menstruation, pregnancy, and parenting: advances and obstacles in reproductive care

In 2018, the United Nations raised the debate on “menstrual poverty” as an aspect that impacts all (cis) women, more severely affecting people in socioeconomic vulnerability, those with disabilities, deprived of liberty, and transgender ones. Menstrual precariousness affects the physical and mental health of people who menstruate since it distances them from public environments, reinforcing stigmas and social exclusions. In this context, the United Nations Children’s Fund (UNICEF) defends the implementation of menstrual education as part of sex education, addressing pregnancy prevention and providing opportunities for body self-knowledge and well-being ^30^.

*Resolution n. 40* ^31^ of the Brazilian Ministry of Women, Family, and Human Rights was published in 2020. It provided for guidelines to promote, protect, and defend homeless people’s human rights. It states that cisgender women and trans men “*demand specific attention regarding their physiological issues, and sanitary pads, contraceptives, gynecological, and obstetric follow-up in prenatal, childbirth, and postpartum should be offered*”. Xavier’s monograph ^30^ analyzes the laws and public policies in Brazil about “*poverty and menstrual dignity*”. Among them, *Municipal Law n. 6,662* of Campo Grande (Mato Grosso do Sul) and the State Menstrual Dignity Program of Paraíba (both from 2021) explicitly included trans men.

Xavier’s investigation showed varying geographic coverage, beneficiaries’ characteristics, and normative particularities, with no federal initiative yet in place. In 2023, the year of the appointment of the first woman to take over the Brazilian Ministry of Health, *Decree n. 11,432* was published, which regulates *Law n. 14,214/2021*. The debate around it, which, in turn, instituted the Program for the Protection and Promotion of Menstrual Health, grew to include the concept of “menstrual dignity” and include “people who menstruate” other than cisgender women as beneficiaries of the program ^30^ ^,^ ^32^. Despite efforts to include diversity in the regulations, other materials need attention beyond inclusive language, as is the case of the *Implementation Guide* of the aforementioned program, which still refers to “women” and the visual identity of which includes photographs of female people, assuming that they are cis women ^33^.

The Plan to Combat Maternal and Childhood Mortality, in line with the 2030 Agenda for Sustainable Development Goals, the guidelines of which were approved by *Resolution n. 42/2018* ^34^, is another document that corroborates the invisibility of transmasculine fatherhood, maintaining the focus of health care on cis and heterosexual women. Some authors highlight the national absence of public policies that provide for comprehensive care for trans men who wish to become pregnant. These gaps lead to legal violations of reproductive rights, family planning and personality, thus causing transphobia and obstetric violence when these individuals seek health services ^10^ ^,^ ^16^ ^,^ ^24^ ^,^ ^35^.

The inclusion of trans men and transmasculine people in obstetric care is essential given the increased visibility of the trans population and the desire for biological paternity of this group. In this context, assisted reproduction provided opportunities for other family configurations ^36^. However, in practice, people still face institutional, symbolic, and economic barriers. The analysis of the regulations that govern assisted reproduction showed, in Federal Council of Medicine *Resolution n. 2,320/2022*, the revocation of previous versions that included transgender people and the persisting use of terms such as “woman” for people with a uterus ^37^ ^,^ ^38^. Moreover, the high costs and the requirement for hormonal interruption make it difficult to access these procedures, especially via SUS, leading many trans people to seek informal alternatives ^15^ ^,^ ^36^. Although difficult to access, professionals and users should be aware of fertility preservation, especially those who intend to undergo surgical procedures and cross-hormone therapy ^39^.

The gestational paternity of trans men and transmasculine people saw progress in legal recognition in 2021, when the STF determined the replacement of the term “mother” with “parturient” in declarations of live births, respecting the gender identity of those who are pregnant ^24^. The benefits of the change included promoting the collection of data for the elaboration of public policies according to parents’ gender ^14^. However, in practice, many users report the lack of safe and inclusive services and criticize discrimination and inappropriate behavior of healthcare providers when accessing health services ^14^. The denial or inefficiency in filling out a document also harm their children because it is impossible to access basic rights such as consultations, vaccination, and education without the document ^35^ ^,^ ^39^.

### Between recognitions, erasures, and political reconstructions

The absence of recognition of the reproductive rights of trans men is noticeable also in materials aimed at managers and healthcare providers, as in the 2018 Brazilian Ministry of Health *Sexual and Reproductive Health: Men as Subjects of Care*. The document is related to the PNAISH. However, it does not include the health needs of trans men, only mentioning them regarding the use of their social name ^22^. In view of this, social movements show their importance in the elaboration of reference materials based on the perspective of their own experiences and health demands. In the same year, the booklet *Health of Trans Men and Transmasculine People* was released, having been organized by the Trans Men Center of the Trans Brazil Network. Its content included fertility, hormones, surgeries, prevention, and obstetrics ^15^.

Despite the discrete historical advances in the reproductive health of transmasculine people and trans men in Brazil, this issue suffered successive blows and setbacks in 2019 (during the mandate of a far-right government) ^15,29.40^. The booklet was removed, revised, and (after a pushback movement), republished under the title *Trans Men: Let’s Talk about the Prevention of Sexually Transmitted Infections (STIs)?* from which several pieces of information were removed due to the absence of scientific basis ^15^ ^,^ ^22^ ^,^ ^40^. In addition to this material, public policies aimed at the trans population underwent dismantling, including the closure of councils and conferences, budget cuts, and the exchange of qualified professionals for ideological agents, compromising fundamental rights ^40^.

Freitas et al. ^41^ highlight the importance of direct participation led by trans people in the formulation and operationalization of public policies, a space often occupied by female identities, such as *travestis*. The emergence and activity of the Brazilian Institute of Transmasculinities ENT#091;Instituto Brasileiro de TransmasculinidadeENT#093; is fundamental in this context. In 2021, its then coordinator Dan Kaio Lemos ^14^ began his studies and actions on transmasculine pregnancy, seeking to document experiences, promote campaigns, and take the debate to scientific events. The following year, given the lack of government data, the project *Mapping of Trans-masculine Demands Living in Brazil* began, investigating sociodemographic information related to pregnancy, the transsexualizing process, and experienced violence ^14^.

Internationally, during the 72nd World Health Assembly in 2019, the WHO removed transsexuality from the list of mental illnesses of the International Classification of Diseases (ICD) - 11th edition, an essential advance for ensuring of sexual and reproductive rights. The decision was made official, but the implementation of the ICD including this change, although in force internationally since January 2022, is expected to be made available in health surveillance systems in Brazil only in January 2027 ^28^ ^,^ ^42^. In Brazil, the change in governance, as of 2023, preceded resumptions and reconstructions, such as the reactivation of the National Conferences on the Rights of Lesbian, Gay, Bisexual, Travesti, and Transsexual Persons and the institution of the Brazilian National Council for the Rights of Lesbian, Gay, Bisexual, Travesti, Transsexual, Queer, Intersex, Asexual, and Other Persons by *Decree n. 11,471/2023* ^43^ ^,^ ^44^.

More advances occurred in 2024: the creation of the Working Group on Rights and Sexual and Reproductive Health by *Resolution n. 1/2024* (which included representatives of the National Council for the Rights of Lesbian, Gay, Bisexual, Travesti, Transsexual, Queer, Intersex, Asexual, and Other Persons) ^45^, the launch of the prenatal booklet for trans people by the *Transgesta* Program at the Climério de Oliveira Maternity Hospital in Salvador ^46^, Brazilian Ministry of Health *Ordinance n. 1,693/2024* (which expanded the coverage of primary care to the trans and nonbinary population, authorizing the performance of exams and procedures compatible with their anatomical characteristics) ^47^, and the presentation of the Health Care Program for the Trans Population, which proposes the monitoring of the trans population throughout their life cycle, including guaranteeing care for their support network and providing for the inclusion of new procedures and the expansion of qualified outpatient and surgical services ^48^.

Finally, despite these legislative and public policy advances, these normative frameworks fail to comprehensively and respectfully guarantee full access to reproductive rights by themselves. The literature and the analyzed documents indicate that a multiplicity of factors condition the realization of these rights, including service organization, health work processes, professional concepts of gender and reproduction, and the sociocultural contexts in which care is produced. Thus, continuing education in health is a relevant element for addressing the invisibility and distancing transmasculine people experience in health services. Rather than “erasing” women from politics, it refers to including all bodies.

This study had some limitations that require consideration. It restricted itself to official documents and academic productions on specific databases, which may have failed to include less accessible state or municipal legislation and non-indexed institutional materials. Additionally, the operational limitations of search systems in legal and governmental databases, which renders the complete traceability of strategies impossible, may have influenced the identification of normative documents, requiring supplementation by secondary sources. Furthermore, its analysis depended on the availability of updated documents, which may have excluded recent regulations.

The results highlight the need for future studies that further the analysis of the implementation of public policies (especially locally) and that explore the concrete experience of trans men and transmasculine people in accessing reproductive services. Investigations into institutional barriers, obstetric violence, and community resistance strategies can support normative changes and more inclusive practices.

Future regulations should move beyond cisheteronormative formulations, broadening the language and scope of sexual and reproductive health policies to include transmasculine individuals. Similarly, revising documents that use exclusively feminine or binary language can help to reduce interpretative ambiguities and strengthen the recognition of family planning, pregnancy, and assisted reproduction as rights for all people with reproductive capacity.

This study emphasizes that healthcare providers should be aware of existing regulations, such as the right to use social names, the legal recognition of transmasculine parenthood, and the expansion of trans health programs. In everyday practice, this implies offering discrimination-free care, recognizing transmasculine people’s pregnancies and menstrual experiences, and providing adequate guidance on fertility and gamete preservation before irreversible medical interventions. The adoption of educational materials produced by the trans population can configure a strategic tool to train more sensitive and prepared health teams.

## Conclusion

The chronological analysis in this review answered its guiding questions. Although Brazil has important legislative frameworks in reproductive health (such as its Federal Constitution, Family Planning Law, and national health policies for women, men, and the LGBT+ population), these regulations were formulated in historical contexts in which the identity categories and specific demands of trans men and transmasculine people were yet to be consolidated in the public and legal debate. However, by remaining without substantive updates, these provisions produce effects of exclusion and invisibility in the contemporary scenario, showing the persistence of a cisheteronormative logic in the structuring of access to reproductive health.

Regarding access to reproductive health, the rights of this group remain fragile, especially in fertilization, gamete preservation, and family planning, which rarely include them explicitly. These services already have limited availability within SUS given their specialized and highly complex nature. However, the lack of the explicit inclusion of trans men and transmasculine people further intensifies existing barriers, reinforcing disparities in access. Even the most recent SUS and professional council regulations, although showing specific advances (such as the recognition of transmasculine parenthood in the *Declaration of Live Birth* and the launch of the *Transgesta* booklet) still face limitations in their implementation, which impacts the concrete effectiveness of the reproductive rights of this group.

This study found no national public policies that specifically and structurally address access to reproductive health services for transgender men and transmasculine individuals, which may contribute to heterogeneous interpretations. This gap reinforces the dependence on isolated initiatives by social movements and professionals engaged in promoting more inclusive care.

Thus, this review highlights important advances in the normative recognition of the sexual and reproductive rights of trans men and transmasculine people in Brazil. However, the transformation of these legal frameworks into effective care practices does not occur automatically, suffering the influence of institutional, formative, organizational, and sociocultural factors. The incorporation of these guidelines into the daily routine of health services demands, among other aspects, confronting normative conceptions of gender, improving professional practices, and strengthening institutional arrangements committed to equitable care. Thus, recognizing and respecting the reproductive experiences of this population constitute central elements to reduce discrimination and promote care in line with the principles of human dignity and gender self-determination.

## Data Availability

The sources of information used in the study are indicated in the body of the article.
