# Supplementary material for: Normative evolution and institutional challenges of reproductive health of trans men and transmasculine people in Brazil: a scoping review
Source: Cad Saude Publica. 2026 Jul 27;42:e00200525. doi: 10.1590/0102-311XEN200525 (PMC13411463; doi:10.1590/0102-311XEN200525)
Supplement: Supplementary Material [file 1678-4464-csp-42-EN200525-s.pdf]

Supplementary Material 1: Preferred Reporting Items for Systematic reviews and Meta-Analyses extension for Scoping Reviews (PRISMA-ScR) Checklist

SECTION	ITEM	PRISMA-ScR CHECKLIST ITEM	#PAGE
**TITLE**			
Title	1	Identify the report as a scoping review.	#1
**ABSTRACT**			
Structured summary	2	Provide a structured summary that includes (as applicable): background, objectives, eligibility criteria, sources of evidence, charting methods, results, and conclusions that relate to the review questions and objectives.	#1
**INTRODUCTION**			
Rationale	3	Describe the rationale for the review in the context of what is already known. Explain why the review questions/objectives lend themselves to a scoping review approach.	#2-3
Objectives	4	Provide an explicit statement of the questions and objectives being addressed with reference to their key elements (e.g., population or participants, concepts, and context) or other relevant key elements used to conceptualize the review questions and/or objectives.	#3
**METHODS**			
Protocol and registration	5	Indicate whether a review protocol exists; state if and where it can be accessed (e.g., a Web address); and if available, provide registration information, including the registration number.	#3
Eligibility criteria	6	Specify characteristics of the sources of evidence used as eligibility criteria (e.g., years considered, language, and publication status), and provide a rationale.	#3-4
Information sources*	7	Describe all information sources in the search (e.g., databases with dates of coverage and contact with authors to identify additional sources), as well as the date the most recent search was executed.	#4
Search	8	Present the full electronic search strategy for at least 1 database, including any limits used, such that it could be repeated.	#4
Selection of sources of evidence†	9	State the process for selecting sources of evidence (i.e., screening and eligibility) included in the scoping review.	#4
Data charting process‡	10	Describe the methods of charting data from the included sources of evidence (e.g., calibrated forms or forms that have been tested by the team before their use, and whether data charting was done independently or in duplicate) and any processes for obtaining and confirming data from investigators.	#5
Data items	11	List and define all variables for which data were sought and any assumptions and simplifications made.	#5
Critical appraisal of individual sources of evidence§	12	If done, provide a rationale for conducting a critical appraisal of included sources of evidence; describe the methods used and how this information was used in any data synthesis (if appropriate).	NA
Synthesis of results	13	Describe the methods of handling and summarizing the data that were charted.	#5
**RESULTS**			
Selection of sources of evidence	14	Give numbers of sources of evidence screened, assessed for eligibility, and included in the review, with reasons for exclusions at each stage, ideally using a flow diagram.	#5-6
Characteristics of sources of evidence	15	For each source of evidence, present characteristics for which data were charted and provide the citations.	NA

Critical appraisal within sources of evidence	16	If done, present data on critical appraisal of included sources of evidence (see item 12).	#NA
Results of individual sources of evidence	17	For each included source of evidence, present the relevant data that were charted that relate to the review questions and objectives.	#5-6
Synthesis of results	18	Summarize and/or present the charting results as they relate to the review questions and objectives.	#5-7
**DISCUSSION**			
Summary of evidence	19	Summarize the main results (including an overview of concepts, themes, and types of evidence available), link to the review questions and objectives, and consider the relevance to key groups.	#7-15
Limitations	20	Discuss the limitations of the scoping review process.	#14-15
Conclusions	21	Provide a general interpretation of the results with respect to the review questions and objectives, as well as potential implications and/or next steps.	#15
**FUNDING**			
Funding	22	Describe sources of funding for the included sources of evidence, as well as sources of funding for the scoping review. Describe the role of the funders of the scoping review.	#16

Supplementary Material 2: Search strategies for scientific databases addressing the normative evolution and institutional challenges related to the reproductive health of trans men and transmasculine people in Brazil, carried out on April 24, 2025.

**BASE**	**STRATEGIES**
MEDLINE/ PubMed	("Transgender Persons"[Mesh] OR "Transgender Persons"[All Fields] OR "Transgender*"[All Fields] OR "Transmasculine"[All Fields] OR "Trans-Masculine" [All Fields] OR "Trans Masculine"[All Fields] OR "Trans Men"[All Fields] OR "Trans male"[All Fields]) AND ("Reproductive Rights"[Mesh] OR "Reproductive Rights"[All Fields] OR "Reproduction Right"[All Fields] OR "Reproduction Rights"[All Fields] OR "sexual rights"[All Fields] OR "Sexual and Reproductive Health"[All Fields]) AND ("Brazil"[Mesh] OR "Brazil"[All Fields] OR "Brazil*"[All Fields])
EMBASE	('transgender'/exp OR 'transgender persons' OR 'transgender*' OR 'transmasculine' OR 'trans-masculine' OR 'trans masculine' OR 'trans men' OR 'trans male') AND ('reproductive rights'/exp OR 'reproductive rights' OR 'reproduction right' OR 'reproduction rights' OR 'sexual rights' OR 'sexual and reproductive health') AND ('brazil'/exp OR 'brazil' OR 'brazil*')
SCOPUS	TITLE-ABS-KEY (transgender* OR transmasculine OR trans-masculine OR "trans masculine" OR "trans men" OR "trans male") AND TITLE-ABS-KEY ("sexual and reproductive rights" OR "reproductive rights" OR "sexual rights" OR "sexual and reproductive health") AND TITLE-ABS-KEY (brazil)
WEB OF SCIENCE	TS=("transgender persons" OR transgender* OR transmasculine OR "trans men" OR "trans masculine" OR "trans-masculine" OR "trans male") AND TS=("reproductive rights" OR "reproduction rights" OR "sexual rights" OR "sexual and reproductive health") AND TS=(brazil)
LILACS	(mh:"Pessoas Transgênero" OR mh:"Transgender Persons" OR mh:"Personas Transgénero" OR mh:"Personnes transgenres" OR tw:"Pessoas Transgênero" OR tw:transgênero OR tw:transgêneras OR tw:"homens trans" OR tw:"Homem Transexual" OR tw:transmasculino OR tw:transmasculinas OR tw:"transgender persons" OR tw:transgender* OR tw:transmasculine OR tw:"trans men" OR tw:"trans masculine" OR tw:"trans-masculine" OR tw:"trans male" OR tw:"personas transgénero" OR tw:transgénero OR tw:transgéneras OR tw:"hombres trans" OR tw:"Hombre Transexual" OR tw:"personnes transgenres" OR tw:transgenres OR tw:"hommes transgenres" OR tw:"Transsexuelles" OR tw:"Transsexuels") AND (mh:"Direitos Sexuais e Reprodutivos" OR mh:"Reproductive Rights" OR mh:"Derechos Sexuales y Reproductivos" OR mh:"Droits procréatifs" OR mh:"Saúde Reprodutiva" OR mh:"Reproductive Health" OR mh:"Salud Reproductiva" OR mh:"Santé reproductive" OR tw:"direitos reprodutivos" OR tw:"direito reprodutivo" OR tw:"direitos sexuais" OR tw:"direito sexual" OR tw:"direitos sexuais e reprodutivos" OR tw:"saúde sexual e reprodutiva" OR tw:"Saúde Reprodutiva" OR tw:"reproductive rights" OR tw:"reproduction rights" OR tw:"sexual rights" OR tw:"sexual and reproductive health" tw:"Reproductive Health" OR tw:"derechos reproductivos" OR tw:"derechos sexuales" OR tw:"derechos sexuales y reproductivos" OR tw:"salud sexual y reproductiva" OR tw:"Salud Reproductiva" OR tw:"Salud de la Reproducción" OR tw:"Salud Reproductora" OR tw:"droits reproductifs" OR tw:"droits sexuels" OR tw:"santé sexuelle et reproductive" OR tw:"Santé reproductive" OR tw:"Santé de la procréation" OR tw:"Santé de la reproduction" OR tw:"Santé Sexuelle et de la Reproduction") AND (mh:"Brasil" OR mh:"Brazil" OR tw:brasil OR tw:brazil OR tw:"brésil")
GOOGLE SCHOLAR	busca 01 → intitle:(“Pessoas Transgênero" OR transgênero OR transgêneras OR "homens trans" OR transmasculino OR transmasculinas) AND intitle:("direitos reprodutivos" OR "direito reprodutivo" OR "direitos sexuais" OR "direito sexual" OR "direitos sexuais e reprodutivos") AND intitle:(Brasil)<br>busca 02→ intitle:(transgender OR transmasculine OR "trans men" OR "trans masculine" OR "trans-masculine" OR "trans male") AND intitle:("reproductive rights" OR "reproduction rights" OR "sexual rights") AND intitle:(Brazil)
